# Field performance of the SD Bioline Malaria Ag *P.f*. rapid diagnostic test among children under five in Nigeria: insights from the 2021 Malaria Indicator Survey

**DOI:** 10.1186/s12936-026-05827-x

**Published:** 2026-02-12

**Authors:** Bisola Olubiyi, Ayodele Alabi, Isaac Isiko

**Affiliations:** 1 School of Public Health, University of Rwanda, Kigali, Rwanda; 2 Usher Institute, University of Edinburgh, Edinburgh, UK; 3 Centre de Recherches Médicales de Lambaréné, Lambaréné, Gabon; 4 Leiden University Center for Infectious Diseases (LU-CID), Leiden University Medical Center, Leiden, The Netherlands; 5 Faculty of Medicine, School of Population and Public Health, University of British Columbia, Vancouver, Canada

**Keywords:** Malaria, SD Bioline, Rapid diagnostic test, Children under five, Nigeria

## Abstract

**Background:**

Malaria remains a leading cause of morbidity and mortality among children under five years of age in Nigeria. While microscopy is the World Health Organization (WHO) gold standard for malaria diagnosis, rapid diagnostic tests (RDTs), such as the Standard Diagnostic Bioline Malaria Antigen *Plasmodium falciparum* (SD Bioline Malaria Ag *P.f.),* are increasingly used in healthcare and field settings. However, recent reports show variable performances of these tests, and evidence on their performance metrics from large, nationally representative Nigerian samples is limited. This study, therefore, aimed to evaluate the performance of the SD Bioline Malaria Ag *P.f.* RDT using a nationally representative dataset.

**Methods:**

We conducted a secondary analysis of the 2021 Nigerian Malaria Indicator Survey (NMIS), a nationally representative household survey that used a two-stage stratified cluster sampling design. Children aged 6–59 months with valid results for both microscopy and SD Bioline Malaria Ag *P.f* were included. The performance metrics for the RDT were calculated against microscopy as the reference standard, accounting for the survey design.

**Results:**

Among the surveyed children, a 9067-unweighted sample with complete RDT and microscopy test results was analyzed (weighted sample, n = 9131). The SD Bioline Malaria Ag *P.f.* demonstrated a sensitivity of 88.6%, a specificity of 75.2%, a positive predictive value (PPV) of 49.5%, a negative predictive value (NPV) of 96.0%, and an accuracy of 78.1%. Cohen’s kappa indicated moderate agreement with microscopy (κ = 0.496), and the overall weighted ROC AUC was 0.816.

**Conclusion:**

SD Bioline Malaria Ag *P.f*. shows high sensitivity and negative predictive value, confirming its reliability for ruling out malaria in children aged 6–59 months in Nigeria. However, moderate specificity and low positive predictive value with a large microscopy-RDT estimated prevalence gap indicate a risk of overestimating malaria prevalence. False negatives may occur, potentially due to P*f*HRP2/3 deletions, test handling, or non-falciparum infections. These findings underscore the need for confirmatory testing where feasible, with the consideration of complementary diagnostic strategies to optimize surveillance and case management, and ongoing postmarketing evaluation of RDT performance.

## Introduction

Malaria remains a major cause of morbidity and mortality among children under five years of age in Nigeria [1], and early and accurate diagnosis is critical for effective treatment and control. While the World Health Organization (WHO) states that microscopy [2] is the gold standard for malaria diagnosis, rapid diagnostic tests (RDTs), such as the Standard Diagnostic Bioline Malaria Antigen *Plasmodium falciparum* (SD Bioline Malaria Ag *P.f*.), are increasingly deployed in field and clinical settings because of their ease of use, rapid results, cost effectiveness[3], and minimal equipment or training requirements [4]. These qualities make them especially valuable in remote areas with minimal manpower and equipment [5].

Conducting these tests in both clinical and field settings is crucial for accurately identifying malaria cases, initiating prompt treatment, and preventing the misdiagnosis of non-malarial febrile illnesses (NMFIs), and to ensure compliance with WHO recommendations of parasite-based diagnosis before treatment [5, 6]. This ensures that each condition is properly identified and treated, thereby reducing prolonged morbidity and childhood mortality. The SD Bioline Malaria Ag *P.f.* RDT detects the *Plasmodium falciparum* histidine-rich protein 2 (*Pf*HRP2) antigen, *Plasmodium falciparum* being the predominant malaria species in Nigeria. Produced by Abbott™, the manufacturer reported a sensitivity and specificity of 99.7% and 99.5%, respectively, for the SD Bioline Malaria Ag *P.f.* RDT [7]. A previous study evaluating different RDTs reported that the SD Bioline Malaria Ag *P.f.* RDT performed better than the other assessed RDTs [8]. This RDT brand is WHO-prequalified, meeting international quality standards for malaria testing, and is approved for use in both field and clinical settings, with operational endorsement by the Nigeria National Malaria Programme for routine case management [9, 10], and subsequent inclusion in national surveys such as the Nigeria Malaria Indicator survey (NMIS) [11].

Malaria diagnosis, especially in Low and middle-income countries (LMICs), remains heavily reliant on *Pf*HRP2-based RDTs such as the SD Bioline Malaria Ag *P.f.* RDT; however, deletions of the *Pf*HRP2 genes have been reported in 57 countries, including in Nigeria [12, 13]. The WHO recommends switching to non-*Pf*HRP2-based RDTs if 5% or more of *Plasmodium falciparum (Pf)* cases in an area are missed by standard *Pf*HRP2 RDTs due to *Pf*HRP2 deletions [9]. Combination RDTs using both *Pf*HRP2 and pan-*Plasmodium* lactate dehydrogenase (pLDH) antigens provide a suitable alternative in areas with a high prevalence of HRP2 deletions[14]. Aside from these combination RDTs, the Research and Development pipeline of alternatives includes point-of-care (POC) haemazoin and nucleic acid detection assays, biosensors, digital microscopy, and AI algorithms, but the majority of these are yet to reach clinical evaluation[15]. Polymerase chain reaction (PCR) methods can also be used to reliably detect malaria, but they are expensive, time-consuming, and require specialized training [2, 6].

Recent studies have reported conflicting RDT results. They report either a high rate of false positives [16] or a high rate of false negatives [17], each with dire repercussions for patient management. While many studies have assessed the performance of various malaria RDTs in other countries [2, 8, 18–21], evidence from Nigeria is limited. The few available studies are limited to smaller hospital facility-based populations [22], but data from large, nationally representative samples in Nigeria remain lacking, and such studies may not fully capture the variability encountered in broader, community-level or routine screening program settings. Evaluating RDT performance under real-world field conditions across diverse populations, health facilities, and environmental contexts is therefore essential for better understanding the true utility, reliability, and potential limitations of RDTs for clinical case management and national malaria control efforts.

Using data from a large, nationally representative cohort of Nigerian children aged 6–59 months, this study evaluated the diagnostic accuracy of the SD Bioline Malaria Ag *P.f.* against microscopy, the gold standard for malaria diagnosis.

## Methods

### Study design and ethical approval

This study conducted a secondary analysis of the 2021 Nigerian Malaria Indicator Survey (NMIS) dataset. The NMIS, conducted collaboratively between the Demographic Health Surveys (DHS), the Nigerian Malaria Elimination Programme (NMEP), and the Nigerian Population Commission (NPC), collects nationally representative malaria-related indicators from children aged 6–59 months by interviewing their mothers. The NMIS survey protocol was reviewed and approved by the National Health Research Ethics Committee of Nigeria (NHREC) and the ICF Institutional Review Board. Informed consent was obtained from all participants before data collection. All personal identifiers were removed from the dataset to ensure confidentiality, and written approval for this secondary analysis study was obtained from the DHS Program [11].

### Sampling and data collection

The survey employed a two-stage stratified cluster sampling design covering all six geopolitical zones, the 36 states, and the Federal Capital Territory. Stratification was performed by state and place of residence (urban or rural) [11, 23]. In the first stage, 568 enumeration areas (195 urban areas and 373 rural areas) were selected from the national sampling frame with a probability proportional to size. In the second stage, 25 households were systematically selected from each cluster, yielding a total sample of 14,185 households, of which 13,887 were occupied, and 13,727 were successfully interviewed [11, 24]. The survey collected demographic, household, and biomarker data, including malaria testing data.

### Malaria testing procedures

Malaria tests were conducted using SD Bioline Malaria Ag *P.f* kits (Abbott Diagnostics, South Korea) and microscopy. The SD Bioline Malaria Ag *P.f.* detects *Pf*HRP2, *Pf* being the predominant malaria species in Nigeria. Capillary blood was obtained from all eligible participants via a finger prick. A small drop of blood was applied to the RDT cassette, followed by the addition of the manufacturer’s buffer solution, and the results were read after 15–20 min. A test was considered positive if both the control line and the test line appeared and negative if only the control line appeared [11].

Microscopy was performed at the African Network for Drug and Device Innovation (ANDI) Centre of Excellence in Lagos on thick and thin blood smears stained with Giemsa. Two independent, trained laboratory technicians read each smear. In cases of discrepant readings, a third senior microscopist adjudicated the final result. Blinding was implemented: readers were unaware of the RDT results and participants’ demographic or clinical data. Microscopy was used to confirm *P. falciparum* infection and to detect other malaria species (*Plasmodium malariae, ovale, and vivax*) when present [11]. This dual approach provides both rapid, point-of-care detection and confirmatory laboratory diagnosis.

### Quality control and treatment

The quality control measures included cross-checking a random subset of RDT results with microscopy and retraining field staff to maintain standardized procedures. Children who tested positive by RDT were treated with artemisinin-based combination therapy, with referral to nearby health facilities where necessary per protocol, e.g., cases deemed to be severe [11].

### Variable coding and categorization

Children aged 6–59 months with valid paired RDT and microscopy results only were included in the analysis, and categorized into the following age groups: 6–8, 9–11, 12–17, 18–23, 24–35, 36–47, and 48–59 months, following the standard groupings applied in the 2021 NMIS to ensure consistency with the original survey report and comparability of malaria prevalence estimates across age groups [11]. Children under six months of age all had missing test results and were excluded, as were those who were age-eligible but had missing RDT or microscopy results. All other variables were coded according to the NMIS standards.

### Data source and statistical analysis

The NMIS Kids Recode (KR) file, which comprised the unweighted sample of children, was considered and merged with the Persons Recode (PR) file using child and household codes to access biomarker results. All statistical analyses were performed using R statistical software, version 4.5.0 [17]. The complex sampling design of the survey, including sampling weights, stratification, and clustering, was accounted for using the *survey* package [18] to obtain nationally representative estimates and appropriate variance estimation. Sampling weights were applied only to children with complete RDT and microscopy result pairs, and the resulting population-representative sample was analyzed as is but rounded for simplicity in reporting. The diagnostic performance of the SD Bioline Malaria Ag *P.f*. RDT was evaluated against microscopy (reference standard) using sensitivity, specificity, positive predictive value (PPV), and negative predictive value (NPV), with 95% confidence intervals (CIs) calculated using survey-adjusted methods. Analyses were further stratified by child age group and geopolitical zone to examine variation in RDT performance across relevant subgroups.

## Results

**Fig. 1:**
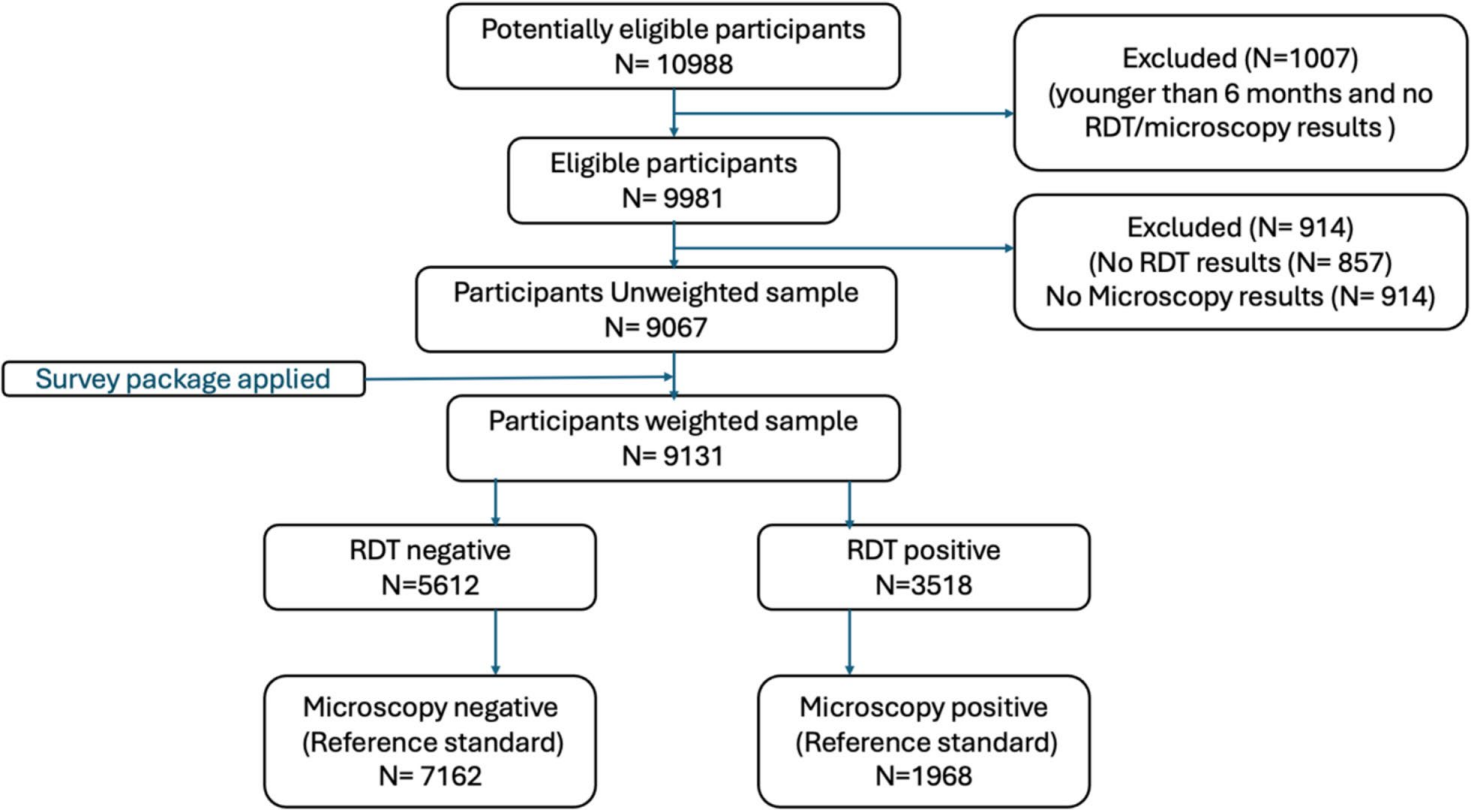
Flow chart illustrating study participant selection and diagnostic test results

The NMIS Kids Recode file contained 10,988 children. After excluding children younger than six months who all had missing test results (n = 1007) and those with missing malaria test results (n = 914), the final unweighted analytic sample comprised 9,067 children aged 6–59 months (Fig. 1). Among these 914 missing malaria test results, all had missing microscopy results, and 857 had missing RDT results. All instances of missing RDT results also lacked microscopy results. After applying survey weights, the weighted analytical sample was 9130.8, rounded to 9,131 for reporting. Minor discrepancies in category totals reflect the individual rounding of the weighted estimates.

A total of 9,131 children aged 6–59 months were included in the analysis, comprising 51.6% males and 48.4% females. The largest proportion of children was aged 48–59 months (24.8%), followed by those aged 36–47 months (22.3%) and 24–35 months (21.6%). Most participants (72.4%) resided in rural areas. The regional distribution revealed that the North West zone accounted for the highest proportion of children (36.3%), whereas the South East zone contributed the smallest share (8.4%). The sample was relatively balanced across household wealth quintiles. The baseline characteristics are summarized in Table 1.

**Table 1:** Baseline characteristics of survey-weighted children aged 6–59 months included in the analysis (N = 9,131)

Characteristic	Category	N*	Percentage (%)
Child’s sex	Female	4423	48.4
	Male	4708	51.6
Child’s age category (months)	6–8	539	5.9
	9–11	436	4.8
	12–17	1028	11.3
	18–23	856	9.4
	24–35	1977	21.6
	36–47	2034	22.3
	48–59	2262	24.8
Residence type	Rural	6613	72.4
	Urban	2518	27.6
Geopolitical zone	North Central	1508	16.5
	North East	1635	17.9
	North West	3316	36.3
	South East	764	8.4
	South South	951	10.4
	South West	958	21.6
Wealth index	Poorest	1985	21.7
	Poorer	1997	21.9
	Middle	1813	19.9
	Richer	1690	18.5
	Richest	1646	18.0
Microscopy result	Negative	7163	78.4
	Positive	1968	21.6
RDT result	Negative	5613	61.5
	Positive	3518	38.5

*Values are weighted using survey sampling weights. Category totals may not sum exactly to the overall sample size due to rounding of individual observations

### Malaria prevalence

**Fig. 2:**
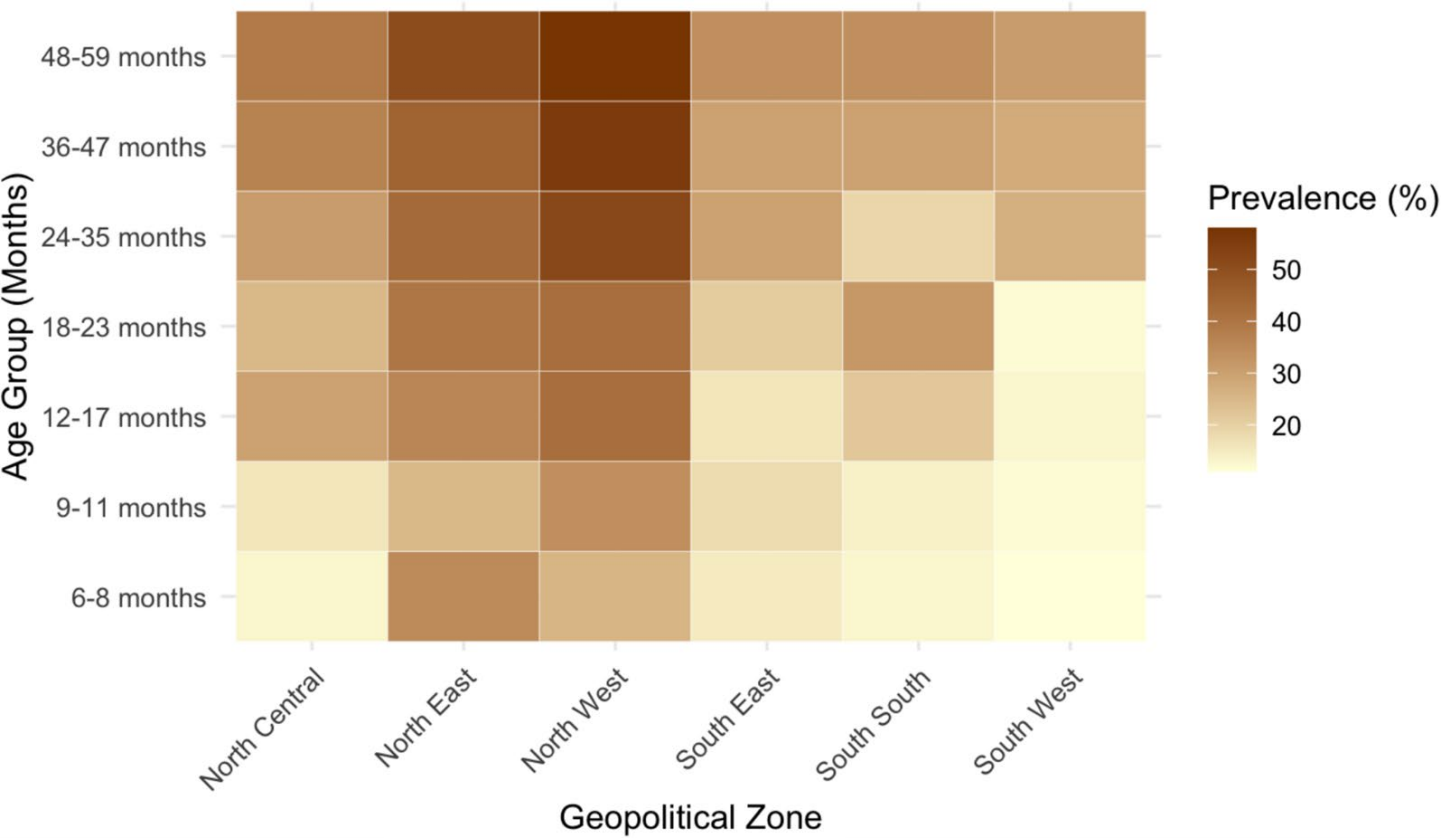
Heat map of survey-weighted SD Bioline Malaria Ag *P.f* RDT-confirmed malaria prevalence by age group and geopolitical zone

Overall malaria prevalence was 21.6% (95% CI, 19.6–23.5%) by microscopy and 38.5% (95% CI, 36.1–41%) by RDT. Notably, malaria prevalence estimated using RDTs was 16.9% points higher than that estimated by microscopy. Age-specific malaria prevalence by RDT varied by zone, and the northern zones experienced both a higher baseline prevalence in younger children and a more pronounced increase as they aged, rising to over 50%, than their southern counterparts (Fig. 2). In contrast, southern zones exhibit a more gradual and less pronounced increase in prevalence with age.

### Plasmodium species

*Plasmodium falciparum* was the most prevalent species (96.7%, 95% CI 95.7–97.7), followed by *P. malariae* (5.8%, 95% CI 4.3–7.3) and *P. ovale* (3.3%, 95% CI 2.1–4.4). No *P. vivax* infections were detected. Mixed infections occurred in 5.8% (95% CI 4.2–7.4) of microscopy-positive children.

### RDT performance metrics

Table 2 presents the cross-classification of rapid diagnostic test (RDT) results with microscopy, the reference standard. Among the 9131 weighted sample of children tested by both methods, 1743 were positive by both RDT and microscopy (true positives), and 5387 were negative by both methods (true negatives). Of the 1968 children with microscopy-confirmed malaria, 225 were negative by RDT, corresponding to a false-negative rate of 11.4%, while 1775 (24.8%) tested positive by RDT but were negative by microscopy (false positives).

**Table 2:** Survey-weighted diagnostic performance of SD Bioline Malaria Ag *P.f* RDT compared with microscopy among children aged 6–59 months

RDT Result	Microscopy negative	Microscopy positive	Total
Negative	5387 (TN)	225 (FN)	5612
Positive	1775 (FP)	1743 (TP)	3518
Total	7162	1968	9130*

*Total reflects weighted counts after applying survey weights to cases with complete paired RDT and microscopy results. Minor discrepancies between the weighted total shown here (9,130) and the overall weighted analytical sample (9,131) are due to rounding of weighted values. No additional observations were included

Compared with microscopy, SD Bioline Malaria Ag *P.f* RDT demonstrated a sensitivity of 88.6% and a specificity of 75.2%. The positive predictive value was 49.5%, whereas the negative predictive value was 96.0%, with an accuracy of 78.1% (Table 3).

**Table 3:** Survey-weighted SD Bioline Malaria Ag *P.f* RDT performance metrics compared with microscopy

RDT performance metrics	Estimate	CI (lower)	CI (upper)
*Diagnostic accuracy*			
Sensitivity	88.5%	86.4%	90.4%
Specificity	75.2%	73.3%	77.1%
Accuracy	78.1%		
*Predictive values*			
Positive Predictive Value (PPV)	49.5%	46.7%	52.4%
Negative Predictive Value (NPV)	96.0%	95.2%	96.6%

### Agreement and diagnostic test evaluation

Based on the two-by-two contingency table (Table 2), Cohen’s kappa coefficient was 0.496 (SE 0.0143), indicating moderate agreement between RDT and microscopy. The weighted McNemar test revealed a significant difference in paired proportions (χ^2^ = 1199.073, *p* < 0.001).

**Fig. 3:**
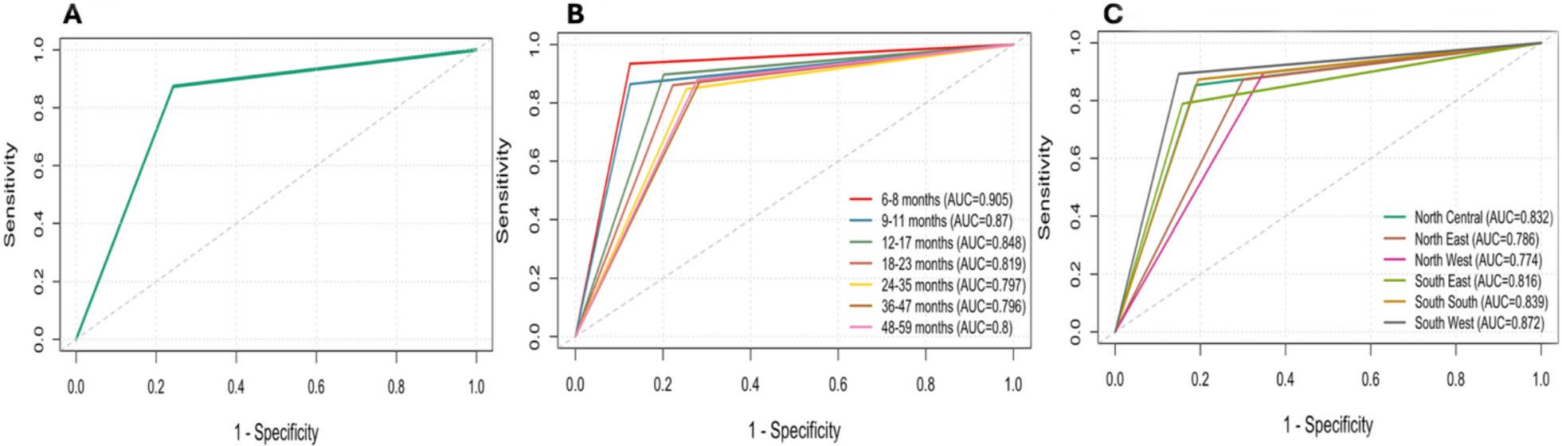
Survey-weighted Receiver operating characteristic (ROC) curves comparing SD Bioline Malaria Ag P.f RDT against microscopy among children 6–59 months in Nigeria (n = 9131). A. Overall weighted ROC curve (AUC = 0.816). B. ROC curves by age group. C. ROC curves by geopolitical zones

Weighted receiver operating characteristic (ROC) curve analysis yielded an overall AUC of 0.816 (95% CI: 0.807–0.825) (Fig. 3a). When stratified by age group (Fig. 3b), the highest AUC was observed among children aged 6–8 months (AUC = 0.905), and the lowest AUC was observed among those aged 24–35 months (AUC = 0.796). Across geopolitical zones (Fig. 3c), RDT had the highest AUC in the South West (AUC = 0.872) and North Central (AUC = 0.832) zones and the lowest in the North West (AUC = 0.774) and South East (AUC = 0.776) zones.

## Discussion

This study demonstrated that the SD Bioline Malaria Ag *P.f* RDT performed modestly under field conditions among Nigerian children aged 6–59 months. The test showed high sensitivity and negative predictive value, although both sensitivity and specificity were lower than manufacturer-reported estimates obtained under controlled conditions [7]. The overall area under the receiver operating characteristic curve (AUC = 0.816) indicates good discriminatory ability. Performance was highest in younger children and declined modestly with age, possibly due to an increased likelihood of false positives in older children from prior infections. Notably, the North West zone, which had the highest malaria prevalence, also had the lowest observed AUC among the zones. Agreement between the RDT and microscopy was moderate (Cohen’s kappa = 0.496), which is expected in high-transmission settings where prevalence and persistent *Pf*HRP2 antigen can influence agreement measures. Overall, the RDT demonstrated good discriminatory performance across age groups and geopolitical zones, with only modest variation between subgroups.

Our findings are consistent with previous evaluations in sub-Saharan Africa, which generally report high sensitivity but variable specificity for the SD Bioline test in both community- and facility-based studies. For example, Wanja et al*.* (Kenya) reported a sensitivity of 94.8% and specificity of 73.3% [2], while facility-based studies in Nigeria have produced similar results. Orimadegun et al*.* (Ibadan, South West Region, Nigeria) reported 95.2% sensitivity, 66.4% specificity, 67.5% PPV, and 94.9% NPV among febrile children aged 3–59 months [22], whereas Adebisi et al*.* reported 94.6% sensitivity, 91.4% specificity, 66.3% PPV, and 98.9% NPV for the CareStart™ HRP2 RDT among children under five years old in the same region [25]. Although these studies were relatively small and facility-based, their diagnostic performance evaluations align closely with findings from this nationally representative cohort of nearly 10,000 children. Collectively, these findings suggest that RDT performance remains relatively robust across diverse study settings, and comparable specificity and PPV indicate that malaria transmission patterns in Nigeria have remained relatively stable over time.

The SD Bioline Malaria Ag *P.f* RDT detects most true malaria cases, but more effectively rules out infection when negative. However, the moderate specificity and relatively low PPV and Cohen’s kappa results highlight the potential for false-positive results [16]. False positives are often attributable to persistent HRP2 antigenemia following recent infections, a phenomenon documented in both Nigeria and Kenya [2, 22], which could lead to overdiagnosis. Such overdiagnosis could lead to unnecessary treatment, overuse of antimalarials, increased risk of drug resistance, and misallocation of limited healthcare resources. Moreover, misdiagnosing nonmalarial febrile illnesses as malaria may delay appropriate treatment, prolong morbidity, or contribute to adverse outcomes.

False-negative results, although less frequent, are of particular clinical concern because they may delay initiating life-saving antimalarial treatment, which could lead to complications from untreated malaria infection [17]. Several factors may have contributed to the false-negative proportion observed in this study, including suboptimal transport or storage conditions of RDTs, low parasite densities, and, although unlikely, operator error [26]. Also, the SD Bioline Malaria Ag *P.f.*RDT is specific to *P. falciparum* and therefore cannot detect other Plasmodium species identified in this study, and these could have accounted for some of the false negatives recorded, although these occurred at low proportions. The contribution of *Pf*HRP2/3 deletions to the proportion of false negatives observed cannot be ruled out. Previous studies in Nigeria have reported marked geographic variability in deletion prevalence, ranging from 17% HRP2 deletions in southwestern Nigeria to 1.6% HRP2/3 double deletions and < 1% single deletions in the southeast, and approximately 9% total HRP2/3 deletions in the North Central zone [13, 27–29]. These false negatives underscore the importance of ongoing postmarketing evaluations, vigilance regarding HRP deletions, and careful interpretation of RDT results in both clinical and surveillance contexts. Future studies and clinical practice should consider combination RDTs targeting both *Pf*HRP and pan-Plasmodium lactate dehydrogenase (pLDH) for more robust detection.

The strengths of this study include the use of a large, nationally representative sample, standardized RDT administration under real-world field conditions, and survey-adjusted statistical analyses. Limitations include the cross-sectional design of the NMIS, which precludes assessment of time-dependent phenomena, particularly whether positive RDT results reflect active infection or persistence of *Pf*HRP2 antigen following recent infection, which may have contributed to false-positive results. Furthermore, microscopy is subject to inter-reader variability, and low-density infections may have been missed. Although dual microscopist slides readings likely reduced this risk, some degree of misclassification cannot be entirely excluded. Nonetheless, microscopy remains the gold standard for malaria diagnosis and is generally preferred over PCR for assessing clinically relevant infection. Finally, the absence of parasite density data limited the evaluation of RDT performance across varying levels of parasitemia. Despite these limitations, the large sample size and robust analytical approach provide strong evidence to support continued evaluation of the performance of SD Bioline Malaria Ag *P.f* in prospective longitudinal studies, while continuing its cautious use in high-burden malaria field and clinical settings.

## Conclusions

The findings from this study highlight the need for caution in interpreting positive SD Bioline Malaria Ag *P.f.* results, and the importance of confirmatory testing in certain contexts to prevent overdiagnosis and unnecessary antimalarial use. Additionally, the RDT false negatives found in this study, particularly in the context of documented *Pf*HRP deletions in Nigeria, further emphasize the importance of confirmatory diagnostic tests. Together, these findings reinforce the importance of continuous postmarketing evaluation of RDTs to ensure diagnostic accuracy under real-world field and clinical conditions. While RDTs remain essential components of malaria control strategies, their results should be interpreted cautiously, and possibly in conjunction with combination RDTs, microscopy, and/or PCR in selected high-transmission settings, to avoid both under- and overdiagnosis and the associated clinical consequences for affected children. Future robust, longitudinal, nationally representative studies will be important to better characterize these diagnostic limitations over time and to support more definitive inferences for policy and practice.

## Data Availability

The data that support the findings of this study are available from The DHS program website upon registration and approval (https://dhsprogram.com/data/dataset_admin/index.cfm).

## Abbreviations

ANDI: African network for drug and device innovation
AUC: Area under the curve
CI: Confidence interval
DHS: Demographic health survey
HRP2: Histidine-rich protein 2
NMEP: Nigerian malaria elimination programme
NMFI: Non-malarial febrile illness
NMIS: Nigeria malaria indicator survey
NPC: Nigerian population commission
NPV: Negative predictive value
PPV: Positive predictive value
RDT: Rapid diagnostic test
ROC: Receiver operating characteristic
SD Bioline Malaria Ag *P.f**.*: Standard diagnostic bioline malaria antigen *Plasmodium falciparum*
WHO: World Health Organization
